# Comparison of learning models to predict LDPE, PET, and ABS concentrations in beach sediment based on spectral reflectance

**DOI:** 10.1038/s41598-023-33207-x

**Published:** 2023-04-17

**Authors:** Faisal Raiyan Huda, Florina Stephanie Richard, Ishraq Rahman, Saeid Moradi, Clarence Tay Yuen Hua, Christabel Anfield Sim Wanwen, Ting Lik Fong, Aazani Mujahid, Moritz Müller

**Affiliations:** 1 Faculty of Engineering, Computing and Science, Swinburne University of Technology, Sarawak Campus, 93350 Kuching, Sarawak Malaysia; 2 Faculty of Science, Thompson Rivers University, 805 TRU Way, Kamloops, BC V2C0C8 Canada; 3 Institute of Sustainable and Renewable Energy (ISuRE), Universiti Malaysia Sarawak, Kota Samarahan, 94300 Sarawak, Malaysia

**Keywords:** Environmental monitoring, Environmental impact

## Abstract

Microplastic (MP) contamination on land has been estimated to be 32 times higher than in the oceans, and yet there is a distinct lack of research on soil MPs compared to marine MPs. Beaches are bridges between land and ocean and present equally understudied sites of microplastic pollution. Visible-near-infrared (vis–NIR) has been applied successfully for the measurement of reflectance and prediction of low-density polyethylene (LDPE), polyethylene terephthalate (PET), and polyvinyl chloride (PVC) concentrations in soil. The rapidity and precision associated with this method make vis–NIR promising. The present study explores PCA regression and machine learning approaches for developing learning models. First, using a spectroradiometer, the spectral reflectance data was measured from treated beach sediment spiked with virgin microplastic pellets [LDPE, PET, and acrylonitrile butadiene styrene (ABS)]. Using the recorded spectral data, predictive models were developed for each microplastic using both the approaches. Both approaches generated models of good accuracy with R^2^ values greater than 0.7, root mean squared error (RMSE) values less than 3 and mean absolute error (MAE) < 2.2. Therefore, using this study’s method, it is possible to rapidly develop accurate predictive models without the need of comprehensive sample preparation, using the low-cost option ASD HandHeld 2 VNIR Spectroradiometer.

## Introduction

Plastics are popular due to their durability, malleable properties, and low-cost manufacturing^1^. However, their overuse and improper disposal methods have led to severe plastic pollution in the environment^2–4^. Plastics that end up in the environment can then through chemical, physical or biological environmental factors break down into smaller fragments known as microplastics (MPs). Several studies reported large numbers of MPs in the marine environment^5–7^. MPs can serve as transport for toxic chemicals, as well as a habitat for harmful microorganisms^8^. They impact and threaten microbial composition, ecosystem health and food chains^9,10^.

However, most of the plastic waste in the marine environment originates from the use of plastics inland^11^. Consequently, microplastic contamination on land is estimated to be 32 times higher than in the oceans^12^. Sources of plastic contamination in the soil environment include sewage sludge which contains primary microplastic (microbeads), fertilizers and personal care products^12,13^. Other sources include landfills and wastewater irrigation^14,15^. Additionally, a vast amount of low-density polyethylene (LDPE) is used for agriculture and for the mulching application^16^. Importantly, these MPs come in contact with soil surfaces from these sources and then seep into subsoils, thus entering the soil environment^6^. They degrade over time into smaller pieces and leak into the groundwater which is used for drinking^17^. Additives in the plastics can leach out which can be harmful to the soil biota^7^. Furthermore, due to plastics’ hydrophobic surface, they absorb other toxicants such as organochlorine pesticides, metals and polychlorinated biphenyls (PCBs)^3^. Lastly, other than absorbing toxicants, the surfaces of soil MPs can harbour microbial pathogens containing antibiotic resistance genes, which can increase the spread of antibiotic resistant microbial diseases^18^.

Despite most MPs being potentially found in soils there is still a distinct lack of research on soil MPs compared to marine MPs^18^. There is even less research on monitoring soil MPs^19^. The need to develop standardized methods of quantifying MPs in soil is well recognized^19–21^. The vast majority of studies used Raman spectroscopy, Fourier Transformed Infra-Red (FTIR) and Pyrolysis–gas chromatography–mass spectrometry (Pyr-GC–MS) to quantify MPs^18,22^. All these methods are time-consuming as the samples must go through density separation to separate out the MPs^23^.

The use of visible-near-infrared (vis–NIR) spectroscopy to identify and quantify MPs has been less explored but successfully used to measure reflectance and predict the concentration of MPs in soil^20,23^. Manley showed that molecules containing X–H chemical bonds i.e. O–H, C–H, give a measurable spectral profile in the vis–NIR spectrum^24^. Thus, through vis–NIR spectroscopy, spectral visualization, establishing relationships between absorption values at specific wavelengths, and appropriate regression model, one can predict and measure the amount of MPs. Through vis–NIR spectroscopy it is also possible to undertake qualitative analyses (classification of plastics) as differences in physical properties are reflected in the spectra.

The potential of machine learning-based microplastic detection and quantification via computer vision and FTIR-spectroscopy has been explored in aquatic ecosystems^25–28^, but there are only a few selected studies on the combination of vis–NIR spectroscopy data and machine learning techniques for microplastic detection in soil^20,23^.

In this study, beach sediment was collected and treated to obtain a treated sediment sample. The soil particles in the sediment were standardized to same size by sieving it through a metal sieve, followed by repeated density separation to remove any MP and impurities in the sediment. Then it was spiked with increasing concentrations of virgin low density polyethylene (LDPE), polyethylene terephthalate (PET) and acrylonitrile butadiene styrene (ABS) micro pellets. The reflectance of the spiked sediment was recorded through vis–NIR spectroscopy (325–1075 nm), and predictive PCA regression and machine learning linear regression models were developed and validated.

## Materials and methods

### Overview of methodological approach

**Figure 1:**
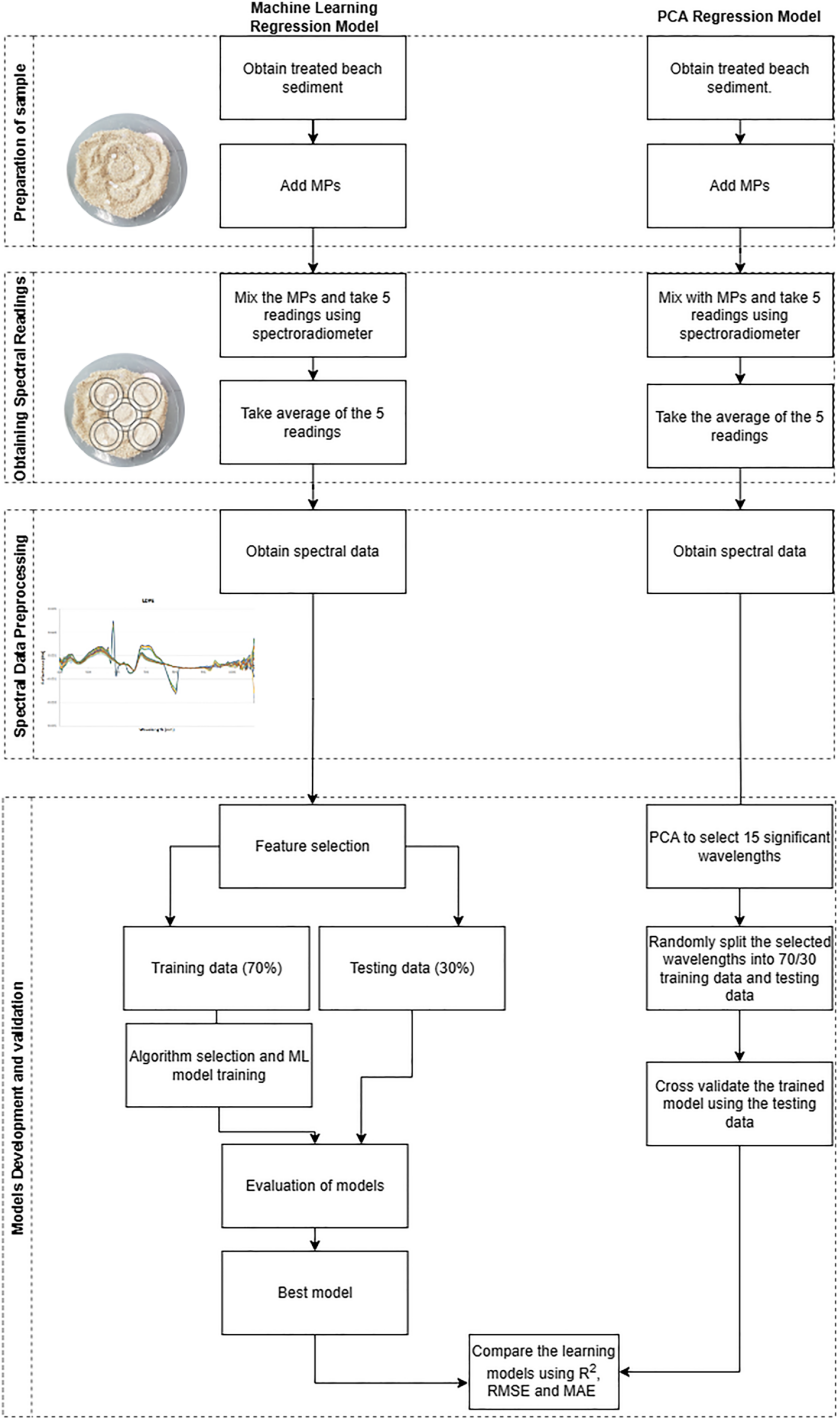
Summary of methodological approach to develop the predictive models. The flow diagram is divided into two sections: machine learning regression model and polynomial regression model.

**Figure 2:**
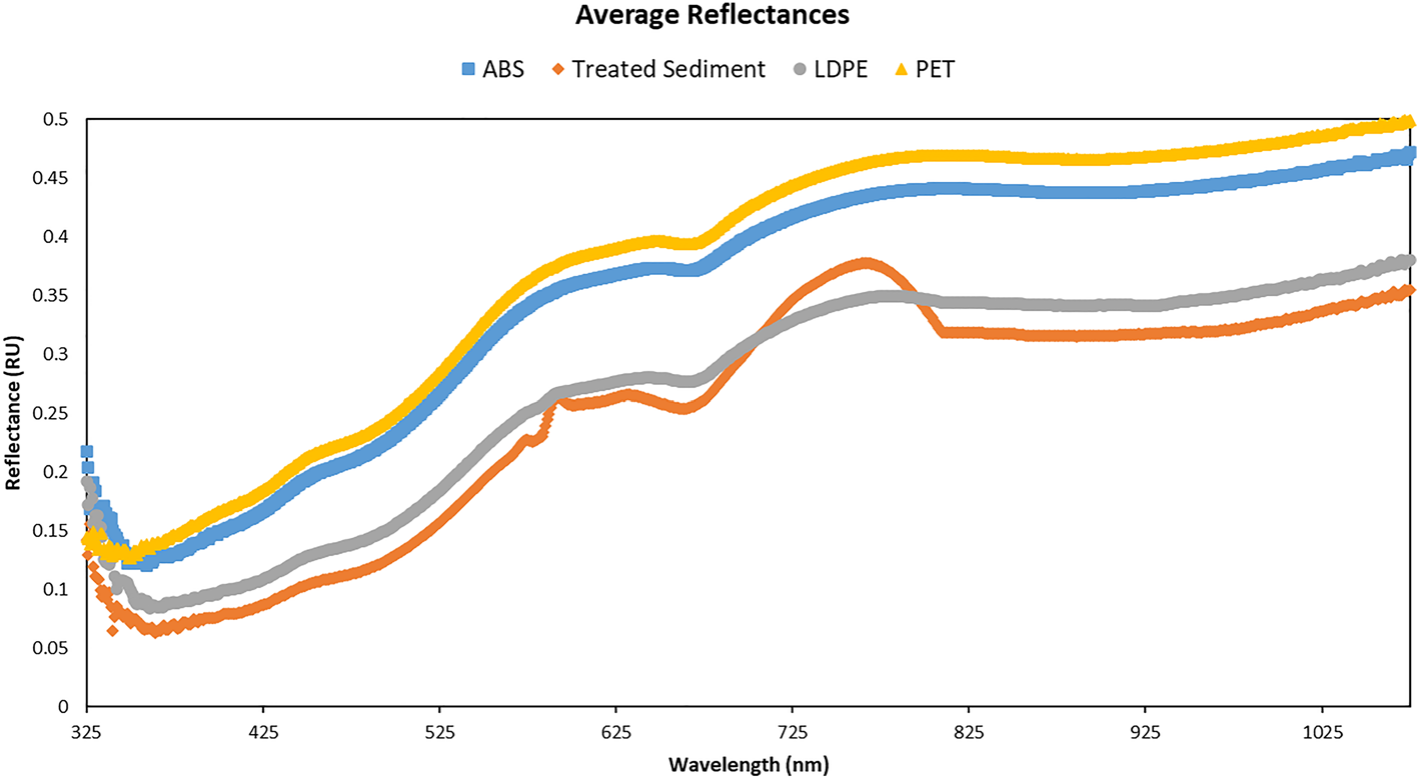
The average reflectance (RU) of ABS (square), treated beach sediment (diamond), LDPE (circle), and PET (triangle) against wavelength (in nm) across all concentrations in treated beach sediment. The average reflectance (RU) of the treated beach sediment without any microplastic is also shown in the figure.

The experiment consists of 4 steps. Figure 1 shows the overview of the methodological approach in this study. Sandy beach sediment was treated and spiked with varying concentrations of LDPE, PET and ABS MPs. The reflectance of the spiked sediment was recorded through vis–NIR spectroscopy, learning models were developed using PCA regression and machine learning linear regression approaches (Fig. 2).

### Sample preparation

#### Collection and pre-treatment of beach sediment

Sandy beach sediment was collected from Damai Beach, Sarawak (1° 45′05.5″ N 110° 18′50.0″ E). A sterile metal spoon was used to collect the top 5 cm layer of beach sediment and transferred into a sterile 1 L glass beaker. The mouth of the glass beaker was securely covered with aluminum foil to prevent contamination from the environment during transport to the laboratory. The removal of MPs and preparation of the beach sediment sample was adapted from He et al.^11^. The beach sediment was sieved using a metal sieve with a mesh size of 1 mm to remove shells, leaves and other large organic substances. 400 g of sieved beach sediment was transferred into a new 1 L glass beaker and density separation was carried out (400 mL of saturated 8.56 molar NaCl, HiMedia, Germany, solution was added into the glass beaker containing the sieved beach sediment). The mixture was stirred for 10 min using a large metal spoon and left overnight, after which the suspension was decanted carefully. Density separation and decanting were repeated twice to ensure all impurities were removed from the beach sediment. To remove excess NaCl after the density separation, the sediment was poured into a 63 µm metal sieve and 1 L of Milli-Q was allowed to run through sediment in the metal sieve. The sediment was then transferred into a glass beaker and allowed to oven-dry at 40 °C for 6 h to obtain a treated beach sediment sample.

#### Reflectance measurements of artificially contaminated beach sediment samples

20 g of the purified beach sediment were transferred onto a watch glass and spiked with virgin LDPE or ABS or PET micro pellets at sequential increments of 0.1% w/w. The microplastic pellets were obtained from Fraunhofer-Institute Karlsruhe, Germany and less than 5 mm in size^29^. ASD HandHeld 2 VNIR Spectroradiometer (Malvern Panalytical, Worcestershire, United Kingdom) was used to record the reflectances in the vis–NIR wavelength range of 325–1075 nm. For each concentration (ranging from 0.1 to 15% w/w), the reflectance was recorded using the contact probe at five different locations, working clockwise from the outer edge of the sample to the center of the sample. Then the average of the 5 readings were used for further analysis. Separate datasets were created for each MP type, where each MP had 46 samples with varying concentrations (0.1–15% w/w), bringing a total of 138 samples studied. Each MP dataset had 230 spectral readings instead of 46 readings since for each concentration there were 5 readings.

### Overview of reflectance processing approaches

After taking the average of 5 readings for each concentration, the datasets of each MP were normalized using the built-in R programming function scale(). The function uses the following formula

$$scaled x= \frac{x- \mu }{\sigma }$$

where $x$ is reflectance value of each wavelength, $\mu$ is the mean reflectance of the wavelength and $\sigma$ is the standard deviation of the reflectance of the wavelength.

After the normalization of datasets, predictive models for the three MP datasets in beach sediment were built through PCA regression approach and machine learning approach. R programming was used for the PCA regression approach while Scikit-Learn from the machine learning approach.

For the PCA regression approach, the packages FactoMineR and factoextra in R programming were used to find 15 most significant wavelengths for each MP dataset through PCA. These 15 significant wavelengths were then randomly split into 70/30 training and testing datasets and then cross-validated. R-squared value (R^2^), root mean squared error (RMSE) and mean absolute error (MAE) were used as models’ performance metrics.

For machine learning, Scikit-Learn software library was implemented in order to identify and select the most significant features (i.e. wavelengths) for each respective microplastic using the feature importance algorithm and Random Regressor algorithm available in the Scikit-Learn library^30^. The feature importance acts as an indicator for each individual contribution of every corresponding feature in a particular classifier^31^. From the regression algorithm selection pipeline, Random Forest (RF) Regressor was used for LDPE, whereas K-nearest neighbor (KNN) Regressor was used for PET and ABS in developing the regression models. The same metrics were (R^2^, RMSE, MAE) generated through this approach to evaluate the models’ performance.

The performance metrics generated by both approaches were then compared.

The following Eqs. (1), (2) and (2) represent the R_2_, RMSE and MAE equations respectively:

1 $$\mathrm{R}^{2} = 1 - \frac{sum squared regression \left(SSR\right)}{total sum of squares \left(SST\right)}$$

2 $$\mathrm{RMSE }= \sqrt{\frac{SS{E}_{w}}{W}}$$

where SSEw = weighted sum of squares, W = total weight of the population.

3 $$\mathrm{MAE }= \frac{{\sum }_{i=1}^{n}\left|{y}_{i}-{x}_{i}{}_{{}}\right|}{n}$$

where MAE = mean absolute error, y_i_ = prediction, x_i_ = true value, n = total number of data points.

### Development of predictive models

#### PCA regression models

This approach, as previously mentioned, utilized the packages FactoMineR and factoextra in R programming to find 15 most significant wavelengths for each MP dataset through PCA. For the LDPE dataset, the correlation matrix was computed through the cor() function. The PCA was then conducted using the princomp() function. The summary() function in R displayed the results from PCA, with the column titled ‘Cumulative Proportion’ observed for the importance of each principal component. To visualize this, fviz_eig() function was used, which displayed the scree plot. Using the scree plot (Supplementary Fig S3) it was determined how many components were needed to explain at least 80% of the total variance in the dataset. The fviz_cos2() function was used to display how much each wavelength contributes to the selected components (arguments for ‘choice’ were set to var, for ‘axes’ was 1:2 and ‘top’ was 15). After the top 15 wavelengths were determined, they were randomly split in 70/30 training and testing datasets. The training dataset was fitted to a regression model using the built-in R function lm(). The formula for the regression model was as follows:–

$$y ={\beta }_{0}+ {\beta }_{1}{x}_{1} +{\beta }_{2}{x}_{2}+\dots +{\beta }_{n}{x}_{n}+ \varepsilon$$

where response variable y is the concentration(w/w), ${x}_{n}$ the predictor variable are the wavelengths from training dataset, ${\beta }_{0}$ is the intercept and ${\beta }_{n}$ is the regression coefficient.

After fitting the model using the training dataset, the diagnostic plots of the model (created using the plot() function) were observed for distribution of residual terms (Supplementary Fig. S5). The testing data set was applied to the trained model using the predict() function in R programming. R-squared value (R^2^), root mean squared error (RMSE) and mean absolute error (MAE) were used as the trained model’s performance metrics. The above steps were repeated for PET and ABS datasets.

#### Machine learning model

**Figure 3:**
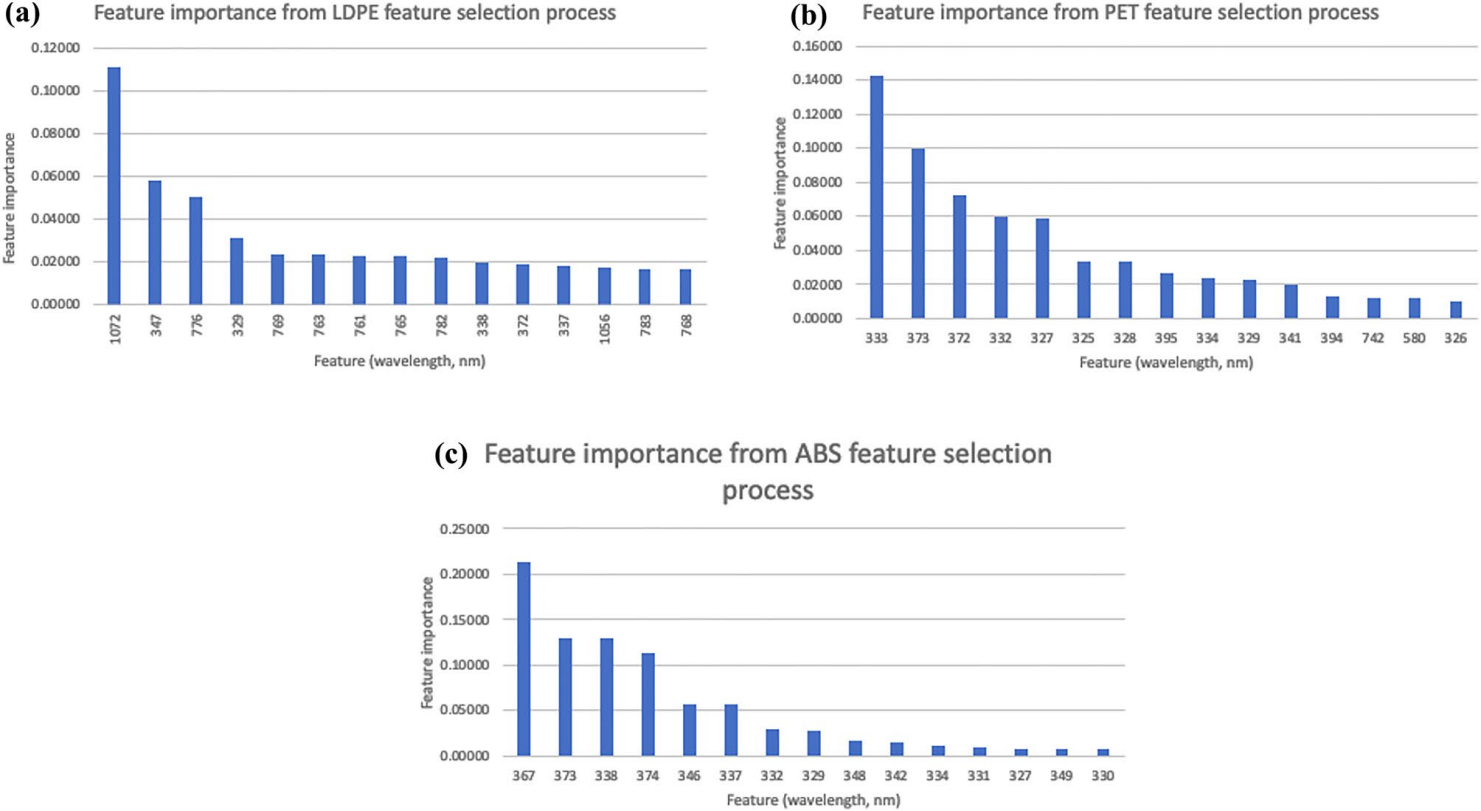
Feature importance of top 15 features from (**a**) LDPE, (**b**) PET and (**c**) ABS data (obtained from feature selection and RF regressor functions in Scikit-Learn). These feature importance graphs rank the wavelengths based on importance in the model development. The highest ranked wavelengths for LDPE, PET and ABS are 1072 nm, 333 nm and 367 nm, respectively. The reflectance values at these wavelength points are applied during the regression models development.

First, the feature importance function and random regressor algorithm from Scikit-Learn library was used to select fifteen features (wavelengths) from the vis–NIR readings of the LDPE, PET and ABS data. The selected features and its importance scores are provided in Fig. 3a–c. The reflectance data from the highest scored wavelength from feature importance function were split into 70% for training and 30% for testing data. Next, a pipeline of regression algorithms with default hyperparameter settings from the Scikit-Learn library was created. The regression algorithms included in the pipeline are included in Supplementary Table S2. Training data from the microplastic samples were iterated into the pipeline and the regression model with the lowest mean squared error (MSE) computed using cross-validation was returned. The details on the MSE computed from the algorithm selection pipeline can be found in Supplementary Table S3. From the regression model selection pipeline, RF Regressor was selected for LDPE data and KNN was selected for PET and ABS data. Then, the training data for each MP sample was used to train the baseline model of the selected algorithms by using default hyperparameter settings. Next, the n_estimators, max_depth and min_samples_split hyperparameters from the RF regressor for the LDPE samples were chosen for tuning. The leaf_size, n_neighbors and p settings for the KNN regressor were selected for tuning for the PET and ABS samples. The best hyperparameter combination settings were determined by using the GridSearchCV function in Scikit-Learn and the hyperparameter-tuned models trained using the training dataset. The models developed were tested using the 30% test data and the performance metrics of these models are summarized in Table 1. The regression graph of predicted vs actual values from the models plotted (Fig. 3a,c). The performance of baseline vs tuned models were compared using the computed MAE, MSE, RMSE and R^2^ values. Learning curves were plotted to ensure the models were not overfitted (Supplementary Fig. S2).

**Table 1:** RMSE, R2, and selected significant wavelengths generated from PCA regression models and machine learning linear models.

Sample	PCA regression models				Machine learning linear regression models			
	RMSE	R^2^	MAE	Significant wavelengths	RMSE	R^2^	MAE	Significant wavelengths
LDPE	2.3	0.83	2.1	326, 325, 327, 336, 335, 328, 329, 330, 332, 331, 334, 333, 337, 342, 532	2.0	0.83	1.3	1072, 347, 776, 329,769, 763, 761, 765, 782, 338, 372, 337, 1956, 783, 768
PET	1.2	0.94	0.95	1068, 1074, 1069, 1064, 1075, 1073, 1044, 1043, 1036, 1027, 1051, 1035, 1045, 1032, 1072	2.7	0.66	1.8	333, 373, 372, 332, 327, 325, 328, 395, 334, 329, 341, 394, 742, 580, 326
ABS	1.2	0.94	0.98	1075, 348, 1066, 1074, 1068, 1061, 1069, 1050, 1065, 1056, 1049, 1067, 1072, 1057, 1071	1.7	0.86	1.1	367, 373, 338, 374, 346, 337, 332, 329, 348, 342, 334, 331, 327, 349, 330

Accuracy metrics for regression models were generated by testing the models on 30% of the test dataset.

## Results

### Microplastic reflectances and regression models

Averaged reflectances recorded using ASD HandHeld 2 VNIR Spectroradiometer across all concentrations and all replicates of each microplastic-sediment sample are shown in Fig. 2 (including an average reflectance of treated beach sediment without any plastic; diamond). The reflectances of PET (triangle), ABS (square) and LDPE (circle) were similar in shape but separated by reflectance intensities with PET recording the highest value and LDPE the lowest. Just the treated beach sediment displayed two overlaps with LDPE around 570 nm and 720–800 nm. The reflectance vs wavelength plots for the three MPs at each concentration (0.1–15%) level are shown in Supplementary Fig. S1.

### Feature selection and machine learning model development

Feature selection using RF Regressor and feature importance algorithms was used to rank important features. The highest ranked feature (wavelength) for each microplastic sample type is used to develop the regression model. Specifically, reflectance data from the 1072 nm, 333 nm and 367 nm were used to develop the regression models for LDPE, PET and ABS samples, respectively. Please refer to Supplementary Table S1 for the feature importance values of each wavelength.

From the machine learning model tuning step, the tuned RF model for LDPE outperformed the baseline model. Meanwhile, there was no improvement in the error metrics of the tuned KNN model for PET. Lastly, the tuned KNN model for ABS outperformed its baseline model. Table 1 summarizes the best regression models for LDPE (baseline RF model), PET (baseline KNN model) and ABS (tuned KNN model). Refer to Supplementary Table S4 for the comparison of the assessment metrics between the baseline vs. tuned models for all MP samples.

### Significant wavelengths selection using PCA

**Figure 4:**
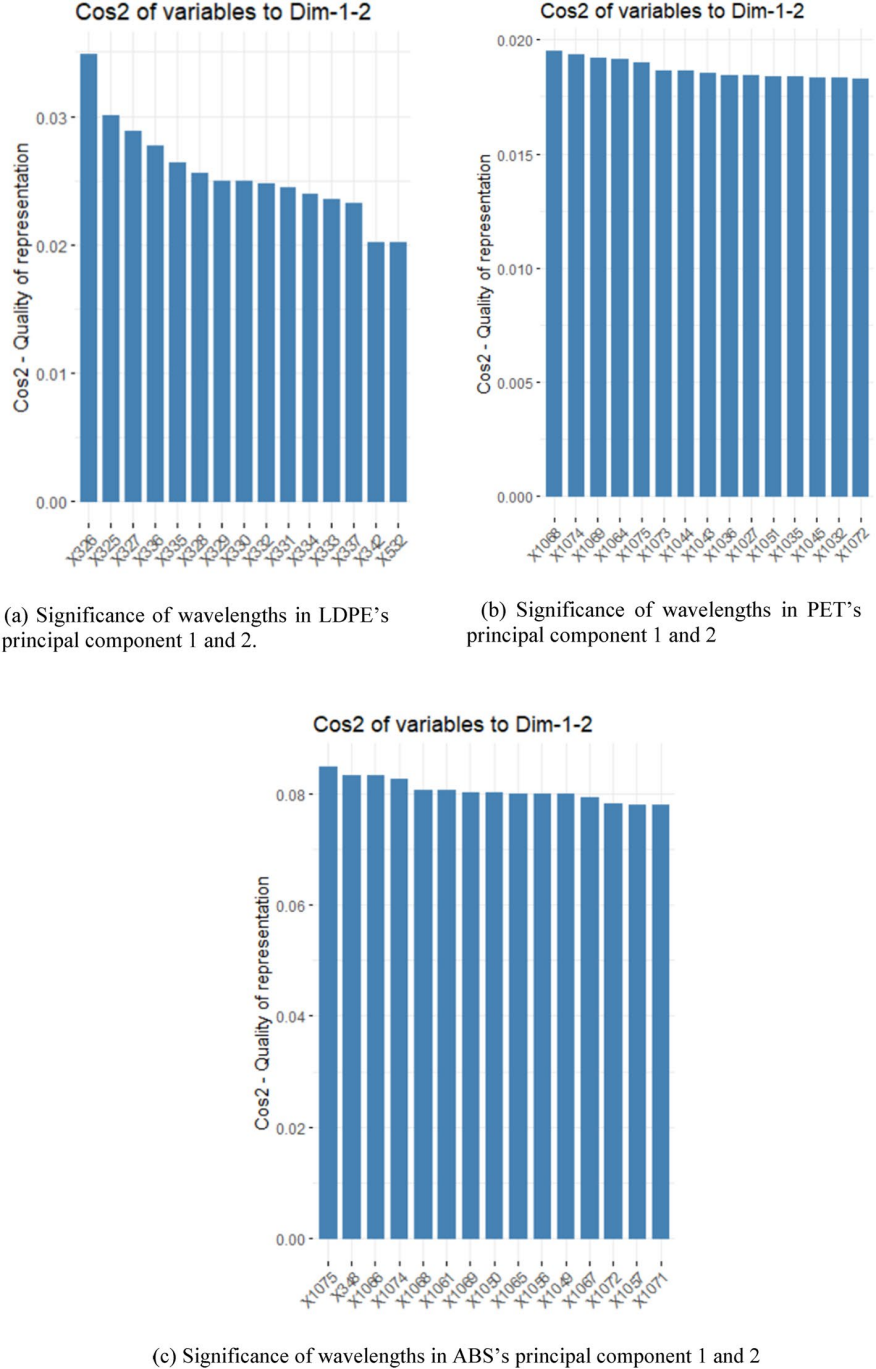
(**a**–**c**) The 15 most significant wavelengths of each microplastic based on square cosine value in the first two principal components, determined using fviz_cos2() function in R programming.

Figure 4 summarizes the 15 most significant wavelengths for the three MPs using PCA. The scree plots (see Supplementary Fig. S3) showed that the first two principal components explained more than 95% of the total variance for all the three MPs. Our dataset for each MP was reduced to 2 principal components without losing much of the data^32^. As mentioned before, the fviz_cos2() function was used in R programming to determine the significance of each wavelength in the given components. A study by Sagar et al. states that in a large multivariate dataset there are many insignificant variables that are not needed for creating the forecasting model^33^.

### Predictive accuracy and significant wavelengths of both methods

Table 1 summarizes the regression plots developed by the two methods. The RMSE values for the PCA regression models and machine learning linear regression models were similar for LDPE and ABS, while RMSE value for PET was better using the PCA regression model. Additionally, the R^2^ values were also better for the PCA regression model. The diagnostic plots (Supplementary Figs. S4, S5) for all the PCA regression models showed a normal distribution of the residuals. It was observed in the QQ-plots for each MP trained model (Supplementary Fig. S5) the points roughly fall on a diagonal line, indicating the residual terms are normally distributed^34^.

Except for LDPE, the significant wavelengths for PET and ABS were mostly in the range of 1020–1075 nm in the PCA approach. On the other hand, the significant wavelengths highlighted using the ML feature importance technique typically fell within the range of 327–374 nm. The highlighted wavelengths indicate that the important wavelengths for regression model prediction are mostly within the noisy range as seen in the scatter plots (see Supplementary Fig. S1). For the ML approach, the hyperparameter tuning also did not result in any significant increase to the performance metrics of the regression model except for the RF regression model. This observation is similar to other reports where studies have shown that the RF is an excellent ML algorithm even without hyperparameter tuning^35^. However, this study shows that the RF and KNN baseline models for the LDPE and PET samples respectively resulted in good model performance while the tuned KNN model had slightly higher performance metrics than its baseline model for the ABS sample. Generally, the hyperparameter tuning did not contribute to significant improvements in any of the regression models. Progressive improvement in the learning curves for all models also indicate that the increase in training data number improves the model’s performance^36^. The learning curve of the KNN model for ABS sample had the smallest gap in between train and validation error curves indicating low model variance and the model had a low variance and lower tendency to overfit. Meanwhile, the baseline RF model developed using the LDPE sample had a slightly larger gap between the train and validation curves than the other models. This indicates the opposite, that is, the model has slightly (1) higher variance and (2) possibility to overfit. Despite the relatively small training data set, the performance metrics indicate the models were well trained, especially for the RF and KNN trained for LDPE and ABS predictions, respectively.

## Discussion

### PCA regression models

RMSE, R^2^ and MAE values (Table 1) were more favorable for the PCA regression plots compared to the machine learning regression plots. Comparing our study with Corradini et al., where the authors used a Bayesian approach to a multilinear regression due to the fact having a higher number of variables than observations, our method tackles this problem by using the PCA approach for variable reduction^23^. The PCA approach in our study is more convenient and quicker than the Bayesian approach. Dai et al. stated that PCA has been widely used for feature selection in spectral datasets and is a better approach when it comes to large spectral datasets which are assumed to have high collinearity^37^. Thus, after PCA, we ensured the selected variables (wavelengths) did not overfit the trained models by observing the models’ diagnostic plots (Supplementary Figs. S4, S5) and the R^2^ values with test datasets (Table 1). However, for further improvement and reducing variables of the training models, conducting stepwise regression after feature selection can fine tune the trained models^37^.

The RMSE value for LDPE’s model is 2.3, indicating that on average, the predicted concentration value deviates from the actual concentration value by 2.3 (% by weight). While RMSE values both for PET and ABS were 1.2. The RMSE values were much better for PET and ABS compared to LDPE. Corradini et al. found the RMSE values for their LDPE and PET models were 0.8 and 1.8 (% by weight)^23^. Even though the RMSE value of our LDPE model is slightly higher than Corradini et al. but the RMSE value of our PET model was lower^23^. Nonetheless, comparing the RMSE values from the study conducted by Corradini et al., we can assume our RMSE values are within acceptable range^23^.

### Machine learning models

A study by Moroni et al. highlighted that the LDPE and PET samples peak at wavelength greater than 1100 nm, the feature importance algorithm applied in this study highlighted different ranges of wavelength importance for the machine learning algorithms to learn^38^. Generally, for PET and ABS, the important features are around the 300 nm range while for LDPE it is around the 300 nm and 700 nm ranges. This indicates that although LDPE and PET samples peak and are better recognized in wavelengths greater than 1100 nm, these wavelengths are not necessarily important for ML models development.

To the best of our knowledge, there are no known studies using ML-based techniques for MPs detection and quantification in soil using vis–NIR data. The closest related study by Corradini et al. reported the application of multilinear regression by regressing the known MPs concentration with absorbance readings at 350–2500 nm for LDPE and PET samples^23^. In Corradini et al., a Bayesian approach was applied to determine the most probable linear regression model^23^. From the same study, the R^2^ values reported were 0.95 and 0.87 respectively in comparison to 0.83 and 0.66 obtained from this study for LDPE and PET, respectively. Although the R^2^ values from Corradini et al. show better regression models, the detection limit was only at 10 g kg^−1^ (1% w/w)^23^. Meanwhile, our study exhibits a higher detection limit of up to 15% w/w of MPs concentration, particularly for LDPE and ABS samples where the R^2^ values are the highest (R^2^ > 0.80). Considering MPs contamination in soil samples are typically beyond 1% w/w detection limit, there is a potential of using vis–NIR and ML linear regression technique for the detection of higher concentration of MPs in soil sediment^20^.

It was observed that some of the significant wavelengths selected by both the models fell in the noisy area of the spectrum, between 325 and 350 nm (Fig. 3). It is possible that the algorithms and PCA mistook the disturbance caused by the noisy data in the spectrum as the most significant variable^39^.

However, in this study, both the approaches generated satisfactory values of R^2^, RMSE, and MAE. Therefore, using this study’s method, it possible to develop accurate predictive models using ASD HandHeld 2 VNIR Spectroradiometer which is a low-cost alternative to the full-range ASD FieldSpec products along with not requiring to use time consuming FTIR procedure and comprehensive sample preparation.

For further studies, differently colored MPs and polymer types can be used to create the regression models as plastics products in our environment have a wide range of color and material.

## Conclusion

Our study explores two approaches in vis–NIR spectroscopy of soil MPs. First, the reflectances of three different virgin microplastics were measured in treated beach sediment, thus standardizing the soil sample. Second, regression models were developed through PCA and machine learning algorithms regression for predicting the MPs in the soil sample.

The results show that the best linear regression models developed for LDPE, PET and ABS using machine learning algorithms resulted in R^2^ values of 0.83, 0.66 and 0.86 with RMSE values of 1.9, 2.7 and 1.7, respectively. The best models developed were from the baseline model except for LDPE whereby hyperparameter tuning resulted in slightly higher accuracy metrics in comparison to its baseline model. The learning curves also indicated that the models' accuracy increased with respect to the training data number suggesting that the ML models can be further improved with the addition of more training data. Previous studies on MPs detection have shown low detection limits. While the detection limit was not quantified in this study, the relatively high accuracy metrics developed for samples up to 15% w/w concentration of MPs, indicates the potential of using this technique to detect MPs with higher detection limit. On the other hand, the PCA regression technique also displayed several advantages. The R^2^ values for LDPE, PET and ABS models were 0.83, 0.94, and 0.44 with RMSE values of 2.3, 1.2, and 1.2, respectively. The performance metrics of these models indicate that it is possible to develop accurate predictive models using the low-cost option ASD HandHeld 2 VNIR Spectroradiometer.

## Supplementary Information

Supplementary Information.

## Data Availability

The datasets generated during and/or analysed during the current study are available from the corresponding author on reasonable request.
