# Supplementary material for: Comparison of learning models to predict LDPE, PET, and ABS concentrations in beach sediment based on spectral reflectance
Source: Sci Rep. 2023 Apr 17;13:6258. doi: 10.1038/s41598-023-33207-x (PMC10110612; doi:10.1038/s41598-023-33207-x)
Supplement: Supplementary file 1 — Supplementary Information. [file 41598_2023_33207_MOESM1_ESM.docx]

# Comparison of learning models to predict LDPE, PET, and ABS concentrations in beach sediment based on spectral reflectance.

Faisal Raiyan Huda^a,d^, Florina Stephanie Richard^a^, Ishraq Rahman^a,b^, Saeid Moradi^d^, Clarence Tay Yuen Hua^a^, Christabel Anfield Sim Wanwen^a^, Ting Lik Fong^a^, Aazani Mujahid^c^, Moritz Müller^a*^

# 8. Supplementary Information


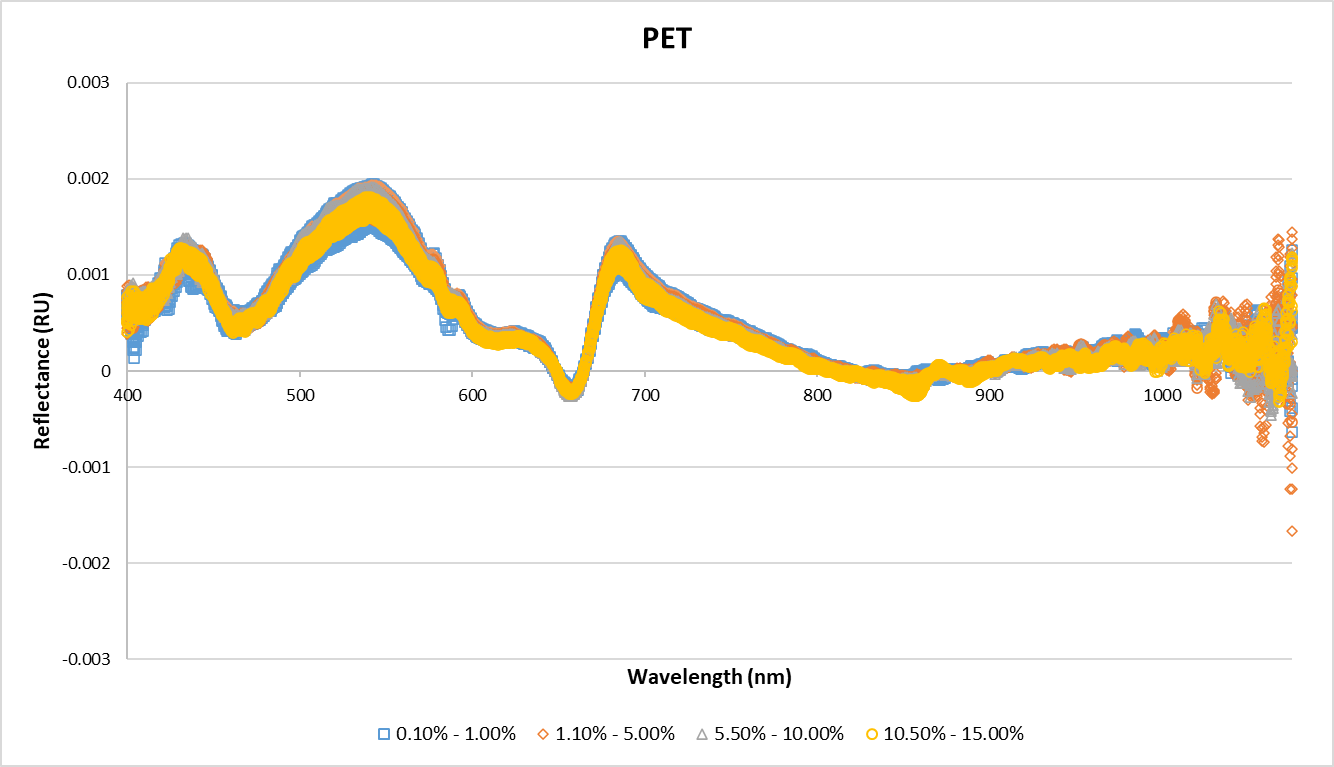


Fig. S1(a): vis-NIR spectra of PET (concentration from 0.1% to 15%) spiked beach sediment sample after preprocessing


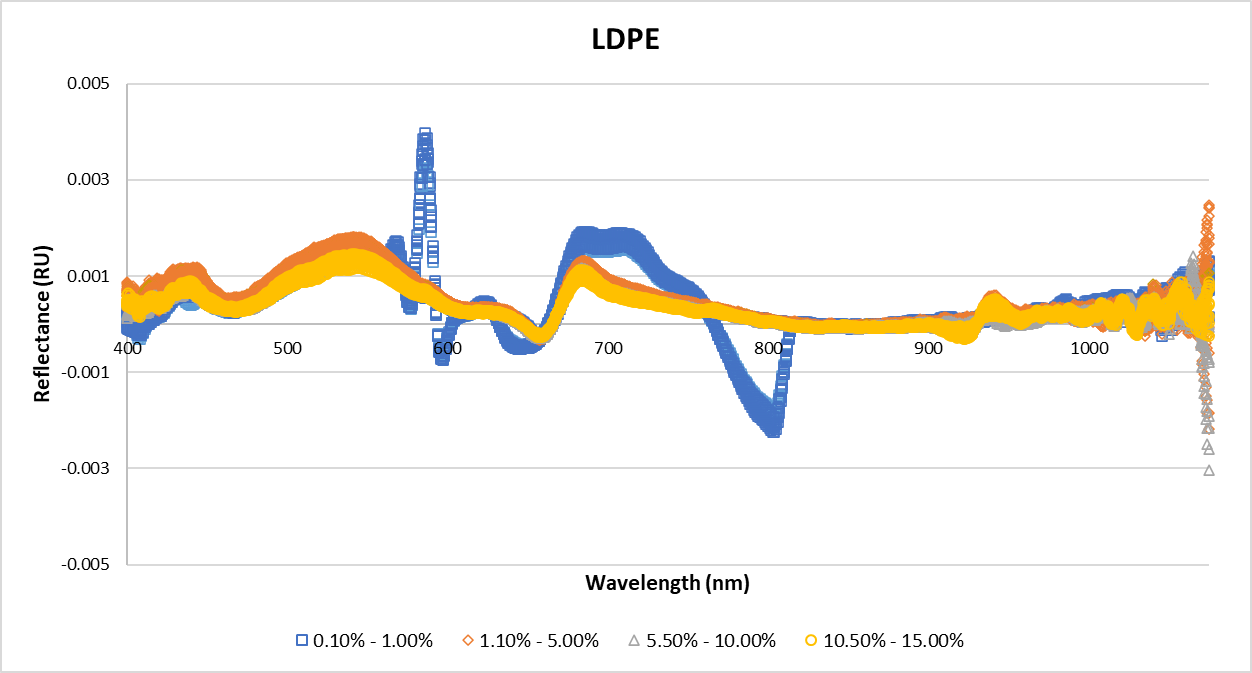


Fig. S1(b): vis-NIR spectra of LDPE (concentration from 0.1% to 15%) spiked beach sediment sample after preprocessing


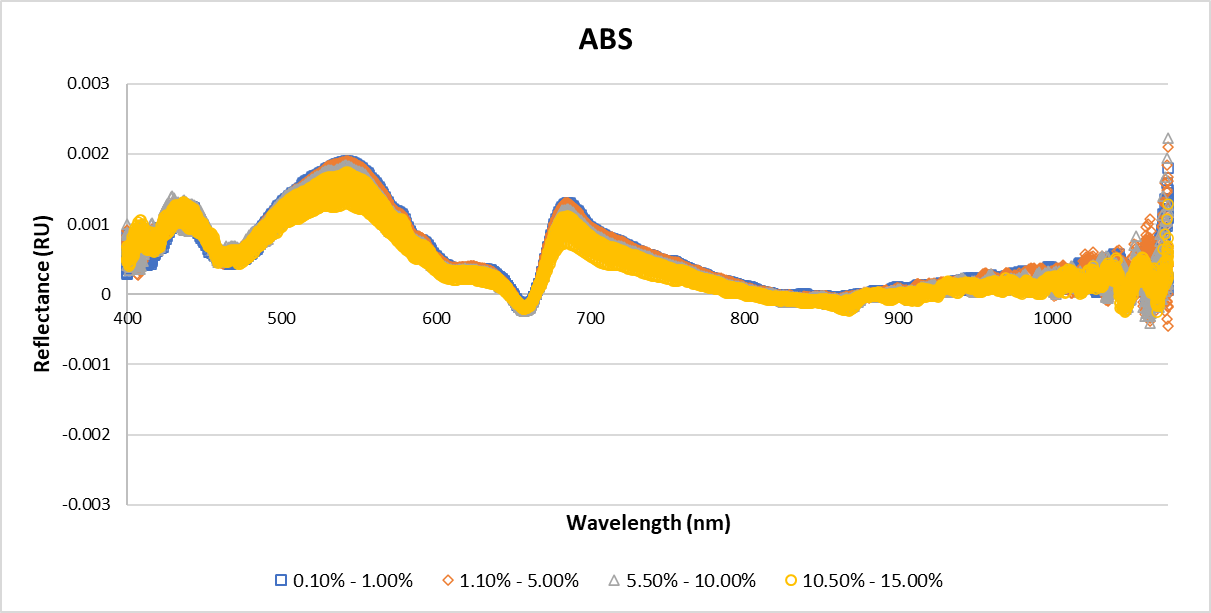


Fig. S1(c): vis-NIR spectra of ABS (concentration from 0.1% to 15%) spiked beach sediment sample after preprocessing

**Fig. S1 (a), (b) & (c): vis-NIR spectra of PET, LDPE & ABS (concentration from 0.1% to 15%) spiked spiked beach sediment sample after preprocessing**

The spectral readings of each MP (with increasing concentration) in the beach sediment plotted against the wavelength range of 400 nm to 1075 nm. ASD Handheld spectroradiometer was used to record the spectral readings after which the readings were preprocessed in OriginPro 2021.

| (a) | 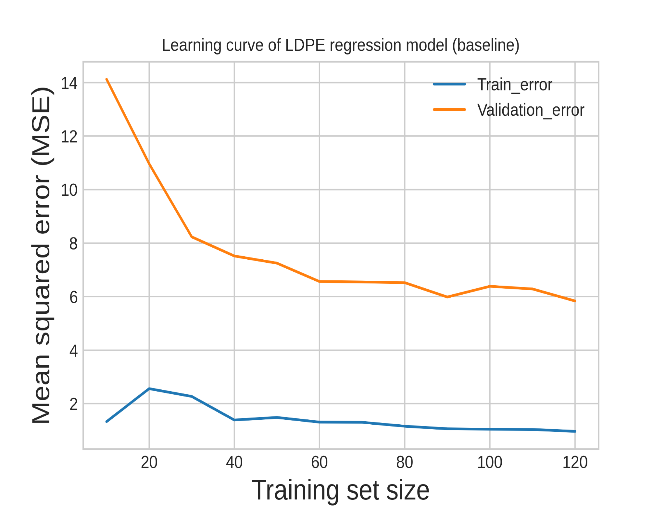 | (b) | 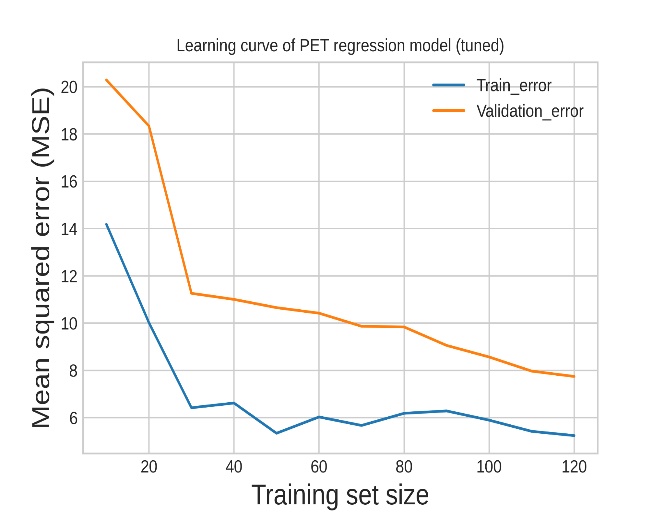 |
| --- | --- | --- | --- |
| (c) | 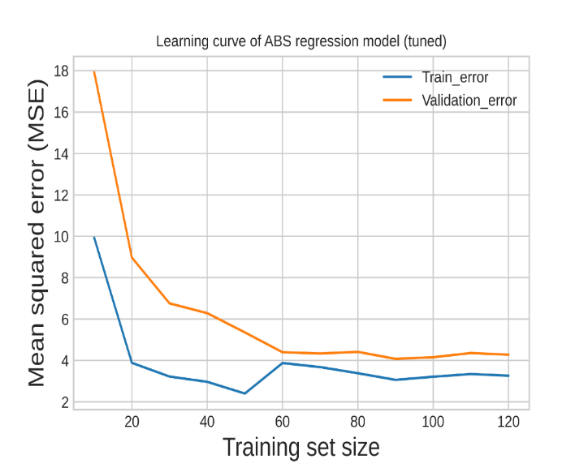 | | |
| **Fig. S2(a) - (c): The learning curves plotted from the tuned RF, tuned KNN and baseline KNN model development respectively.** These curves indicate the validation and training errors of the ML models developed decreases with the increase in the training data number, Narrow gap between the train and validation error curves indicate that the ML model developed has low variance while larger gap indicates otherwise. From these graphs, the baseline RF model has slightly higher variance than the tuned KNN models for both PET and ABS. | | | |


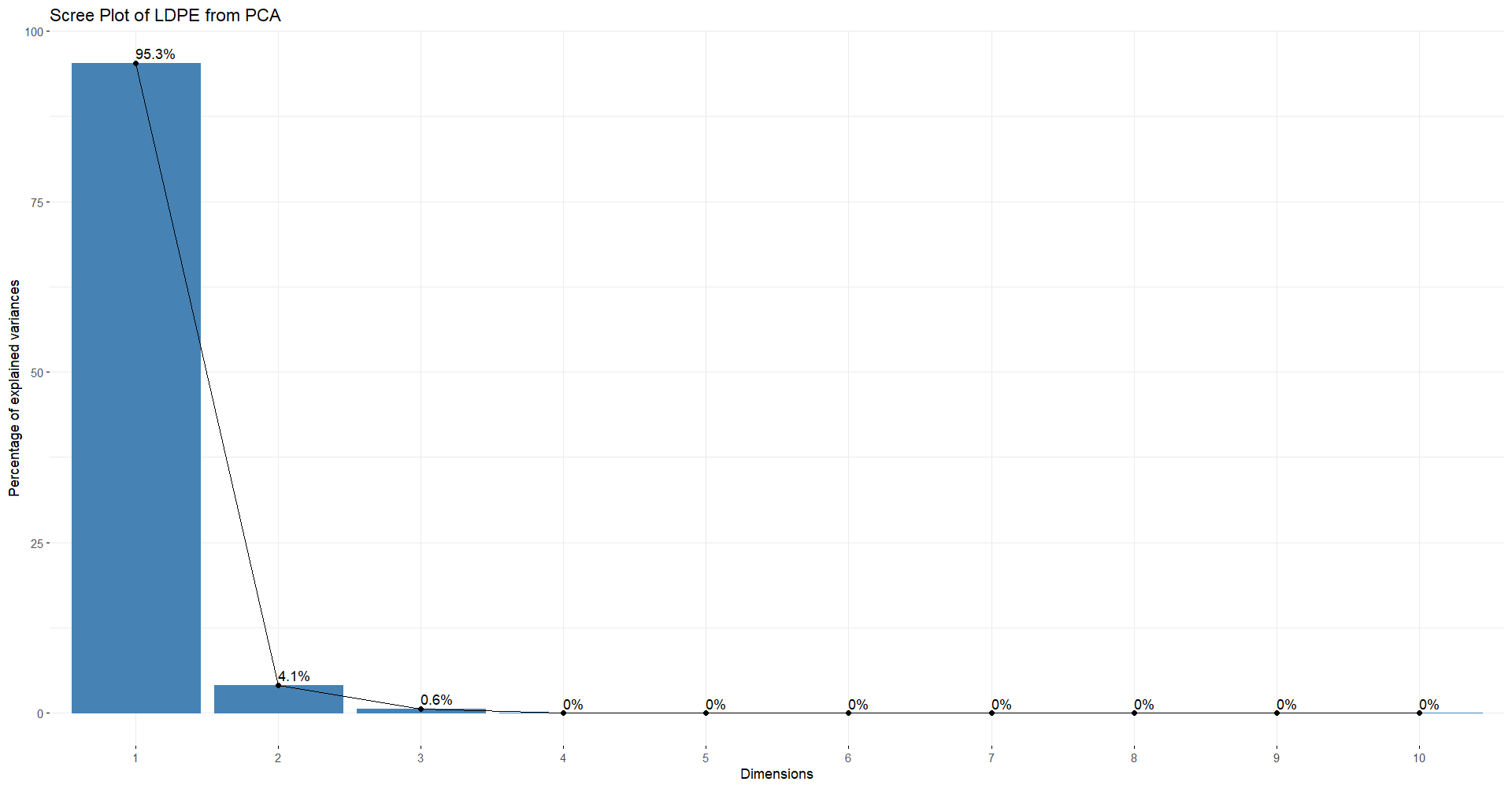


Fig. S3 (a): Scree plot of LDPE


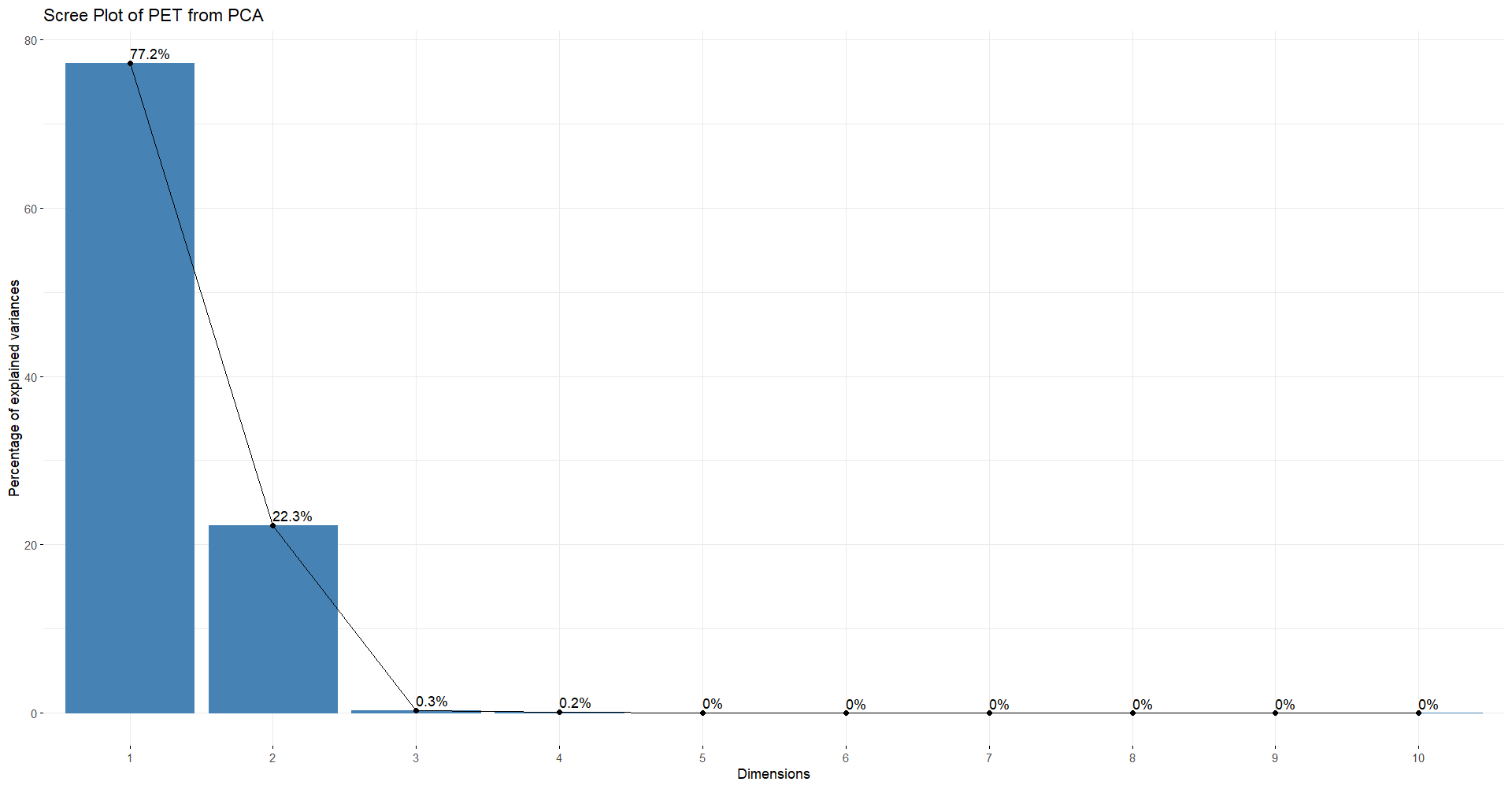


Fig. S3 (b): Scree plot of PET


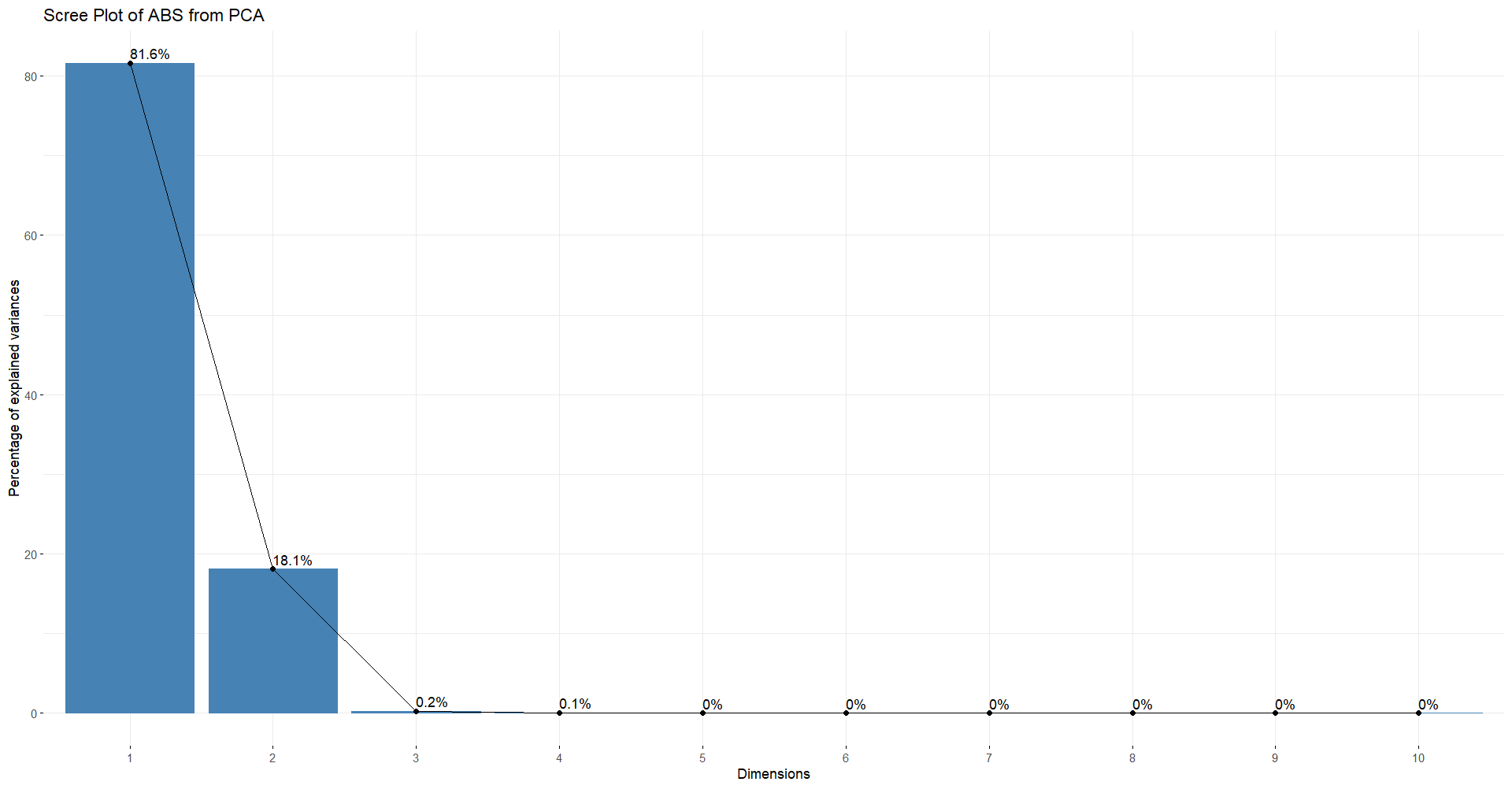


Fig. S3 (c): Scree plot of ABS

**Fig. S3 (a), (b) & (c): Scree plot for each MP in the beach sediment from PCA**. The scree plots visualized the significance of each principal component.

1. Feature selection

**Table S1: Selected features and the importance values**

| **LDPE** | | **PET** | | **ABS** | |
| --- | --- | --- | --- | --- | --- |
| Feature (wavelength) | Importance | Feature (wavelength) | Importance | Feature (wavelength) | Importance |
| 1072 | 0.11 | 333 | 0.14 | 367 | 0.21 |
| 347 | 0.058 | 373 | 0.1 | 373 | 0.13 |
| 776 | 0.051 | 372 | 0.072 | 338 | 0.13 |
| 329 | 0.032 | 332 | 0.06 | 374 | 0.11 |
| 769 | 0.023 | 327 | 0.059 | 346 | 0.058 |
| 763 | 0.023 | 325 | 0.034 | 337 | 0.057 |
| 761 | 0.023 | 328 | 0.033 | 332 | 0.03 |
| 765 | 0.023 | 395 | 0.027 | 329 | 0.027 |
| 782 | 0.022 | 334 | 0.024 | 348 | 0.017 |
| 338 | 0.02 | 329 | 0.023 | 342 | 0.015 |
| 372 | 0.019 | 341 | 0.02 | 334 | 0.011 |
| 337 | 0.019 | 394 | 0.013 | 331 | 0.0087 |
| 1056 | 0.017 | 742 | 0.012 | 327 | 0.0086 |
| 783 | 0.017 | 580 | 0.012 | 349 | 0.0084 |
| 768 | 0.017 | 326 | 0.01 | 330 | 0.0076 |

1. Algorithm selection

**Table S2: Regression algorithm from Scikit-learn included in the algorithm selection pipeline.**

| Regression algorithm | Acronyms |
| --- | --- |
| Lasso | LASSO |
| LGBMRegressor | LGBM |
| LinearRegression | LR |
| ElasticNet | EN |
| DecisionTreeRegressor | CART |
| KNeighborsRegressor | KNN |
| GradientBoostingRegressor | GBM |
| SVR | SVR |
| KernelRidge | KRidge |
| SGDRegressor | SGD |
| RandomForestRegressor | RF |
| BayesianRidge | BRidge |

**Table S3: MSE and SD from the algorithm selection. Highlighted are algorithms with best performance metrics.**

| Algorithm | **LDPE** | | **PET** | | **ABS** | |
| --- | --- | --- | --- | --- | --- | --- |
|  | MSE | SD | MSE | SD | MSE | SD |
| LASSO | 23 | 1.7 | 16 | 1.2 | 27 | 26 |
| LGBM | 6.1 | 1.6 | 8.2 | 2.4 | 23 | 20 |
| LR | 960000 | 1800000 | 8.1 | 1 | 18 | 13 |
| EN | 22 | 1.7 | 13 | 0.51 | 23 | 24 |
| CART | 11 | 1.7 | 13 | 1.3 | 31 | 29 |
| KNN | 8.7 | 2.5 | 6.9 | 2.5 | 15 | 11 |
| GBM | 6.5 | 0.96 | 7.4 | 0.95 | 22 | 22 |
| SVR | 24 | 4.8 | 8.8 | 2.4 | 16 | 15 |
| KRidge | 51 | 7.3 | 37 | 3.3 | 35 | 25 |
| SGD | 22 | 1.3 | 7.9 | 1.2 | 19 | 16 |
| RF | 6 | 1.6 | 8.1 | 1.6 | 21 | 22 |
| BRidge | 59 | 64 | 7.8 | 1.2 | 19 | 16 |

**Table S4: Comparison of the baseline vs tuned regression models.**

|  | Baseline | Tuned |
| --- | --- | --- |
| LDPE (RF) | | |
| MAE | 1.3 | 1.3 |
| MSE | 3.5 | 3.8 |
| RMSE | 1.9 | 1.9 |
| R2 | 0.84 | 0.83 |
| PET (KNN) | | |
| MAE | 1.8 | 1.8 |
| MSE | 7.4 | 7.4 |
| RMSE | 2.7 | 2.7 |
| R2 | 0.66 | 0.66 |
| ABS (KNN) | | |
| MAE | 1.1 | 1.1 |
| MSE | 2.9 | 2.4 |
| RMSE | 1.7 | 1.6 |
| R2 | 0.86 | 0.88 |


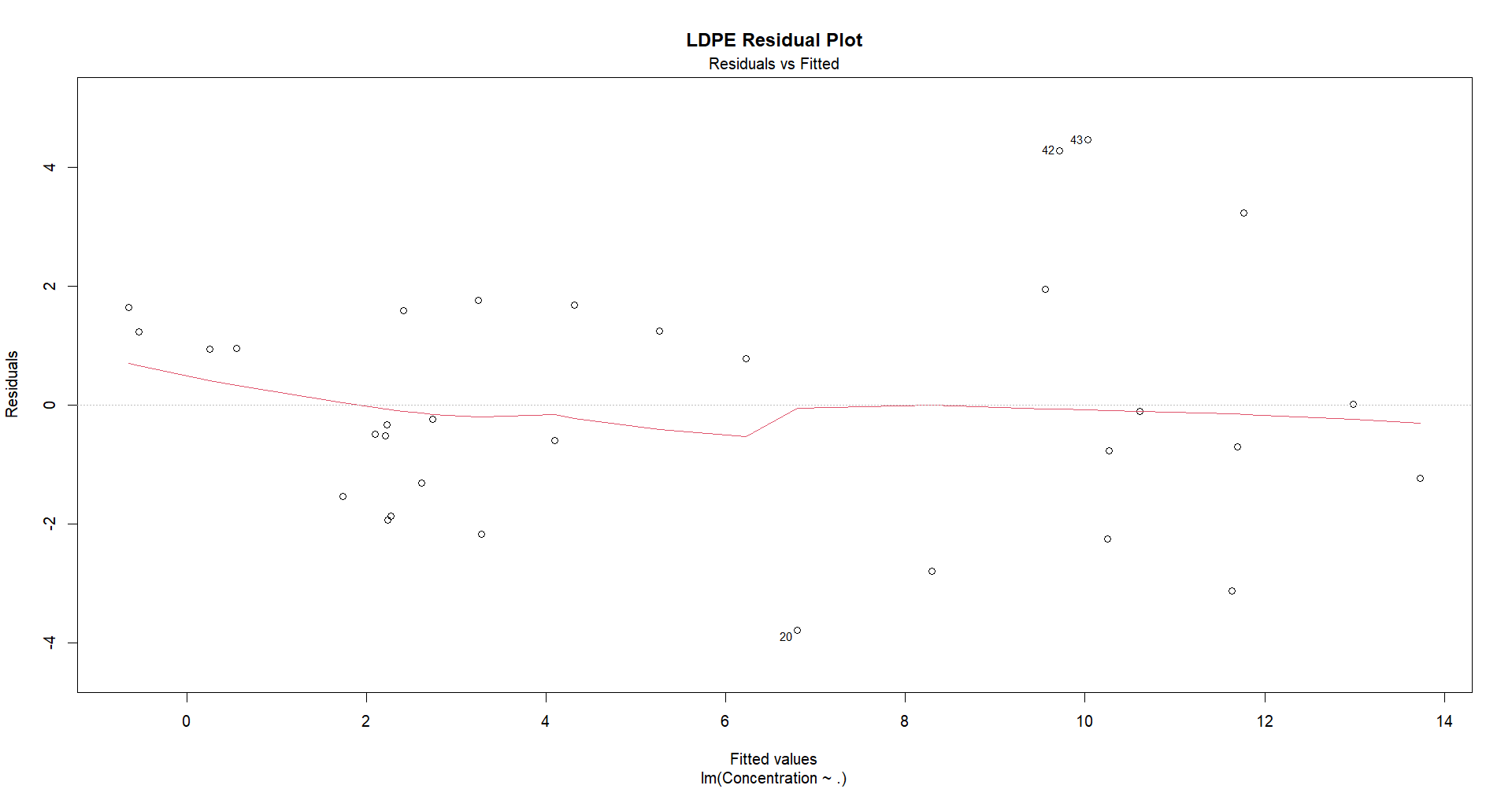


Fig. S4 (a): Residual vs Fitted Plot of LDPE


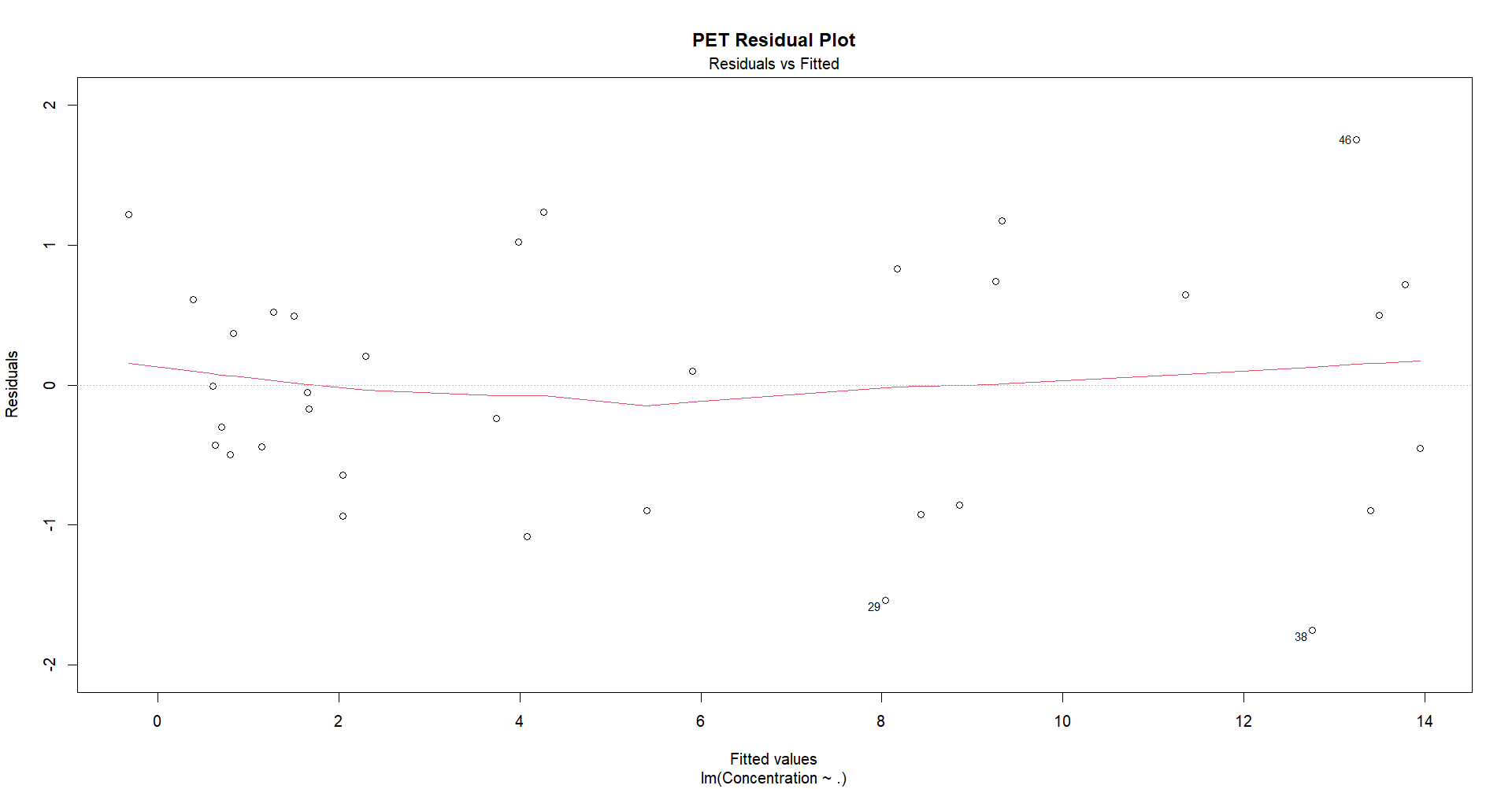


Fig. S4 (b): Residual vs Fitted Plot of PET


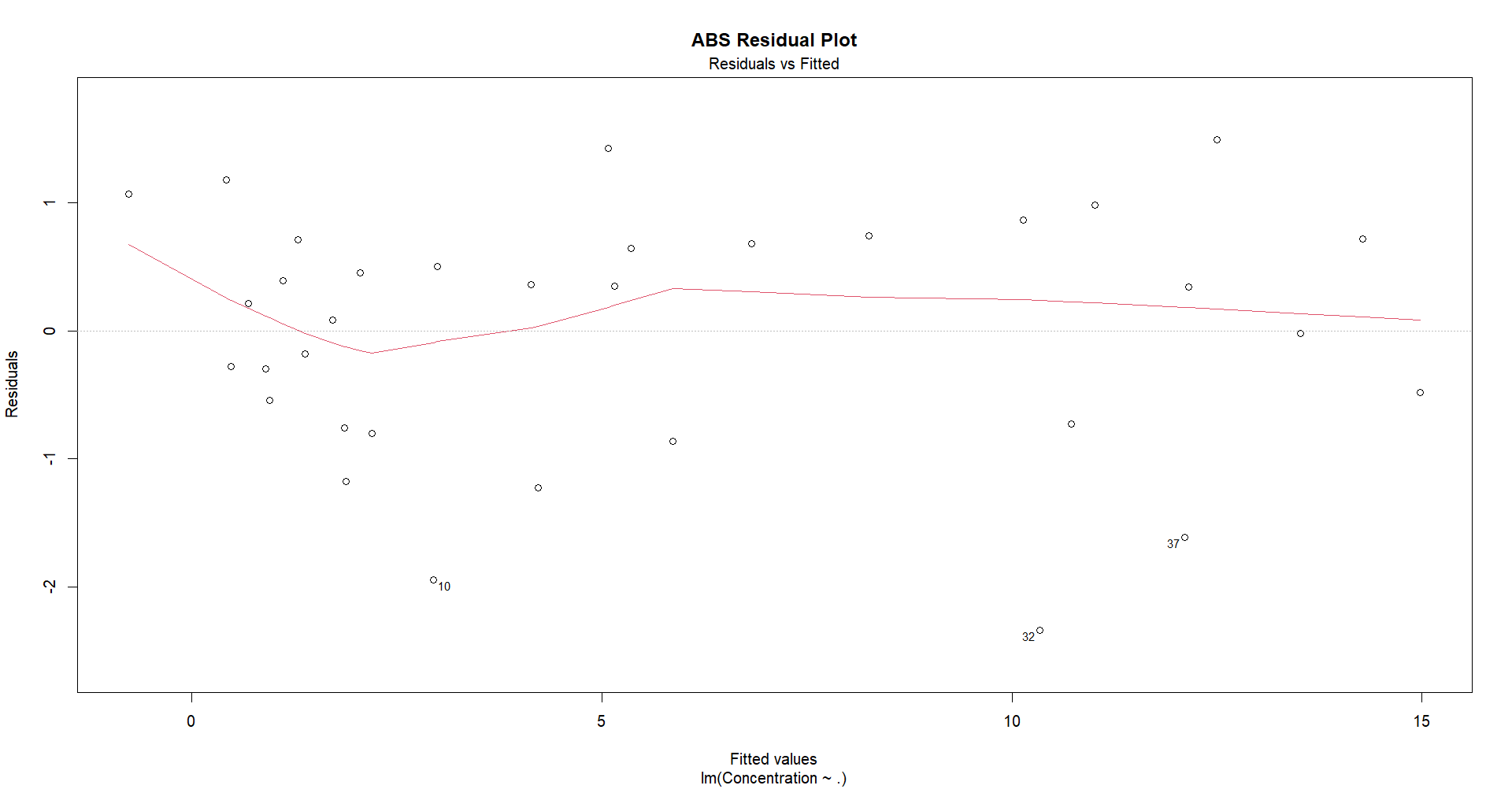


Fig. S4 (b): Residual vs Fitted Plot of ABS

**Fig. S4 (a), (b) and (c): The Residual vs Fitted plots of the trained models using the significant wavelengths of each microplastic dataset.** The Residual vs Fitted helps us to observe if there are any non-linear patterns. If it is roughly horizontal, we can assume it’s a linear pattern.


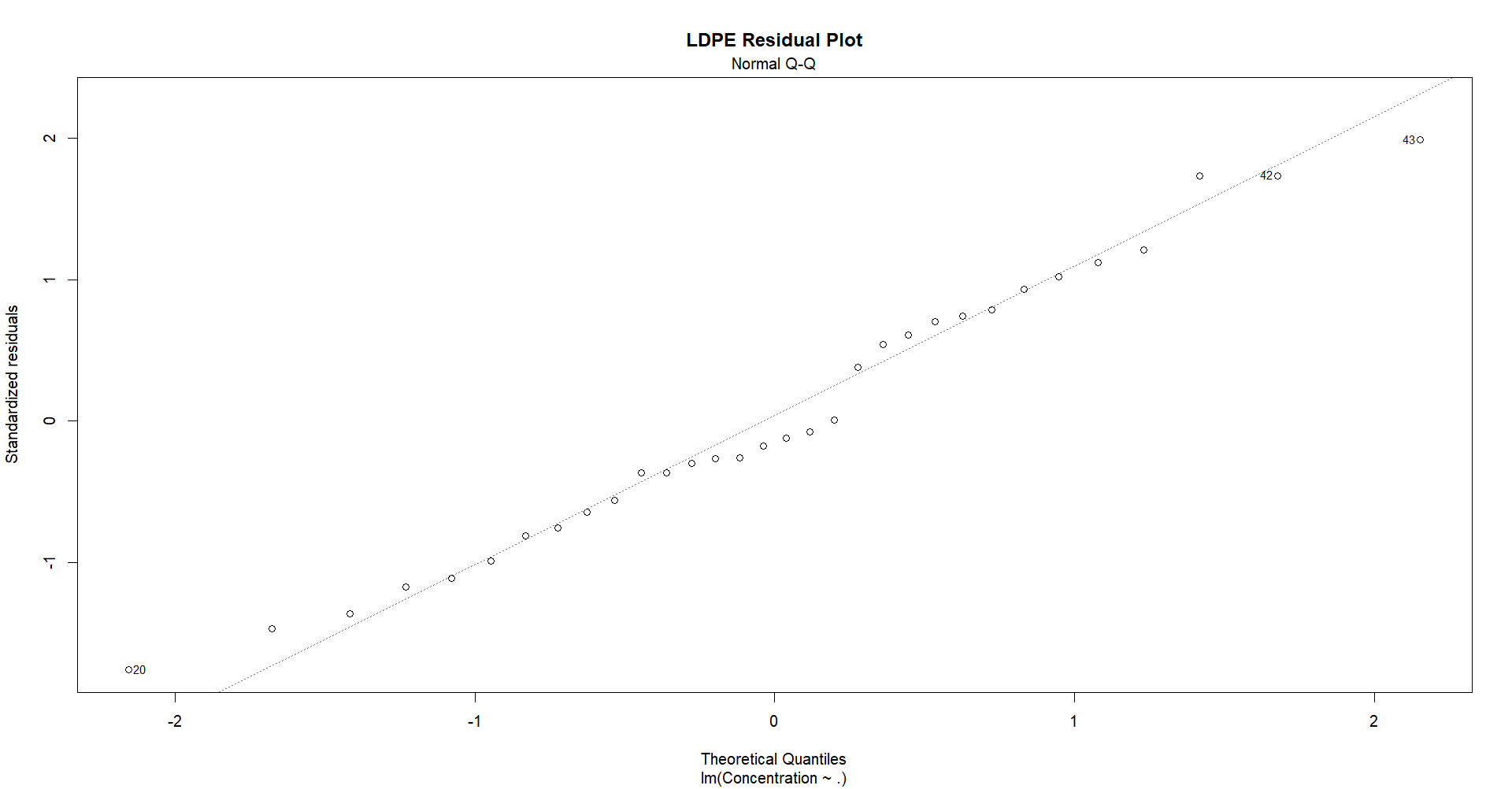


Fig. S5 (a): Normal Q-Q plot of LDPE


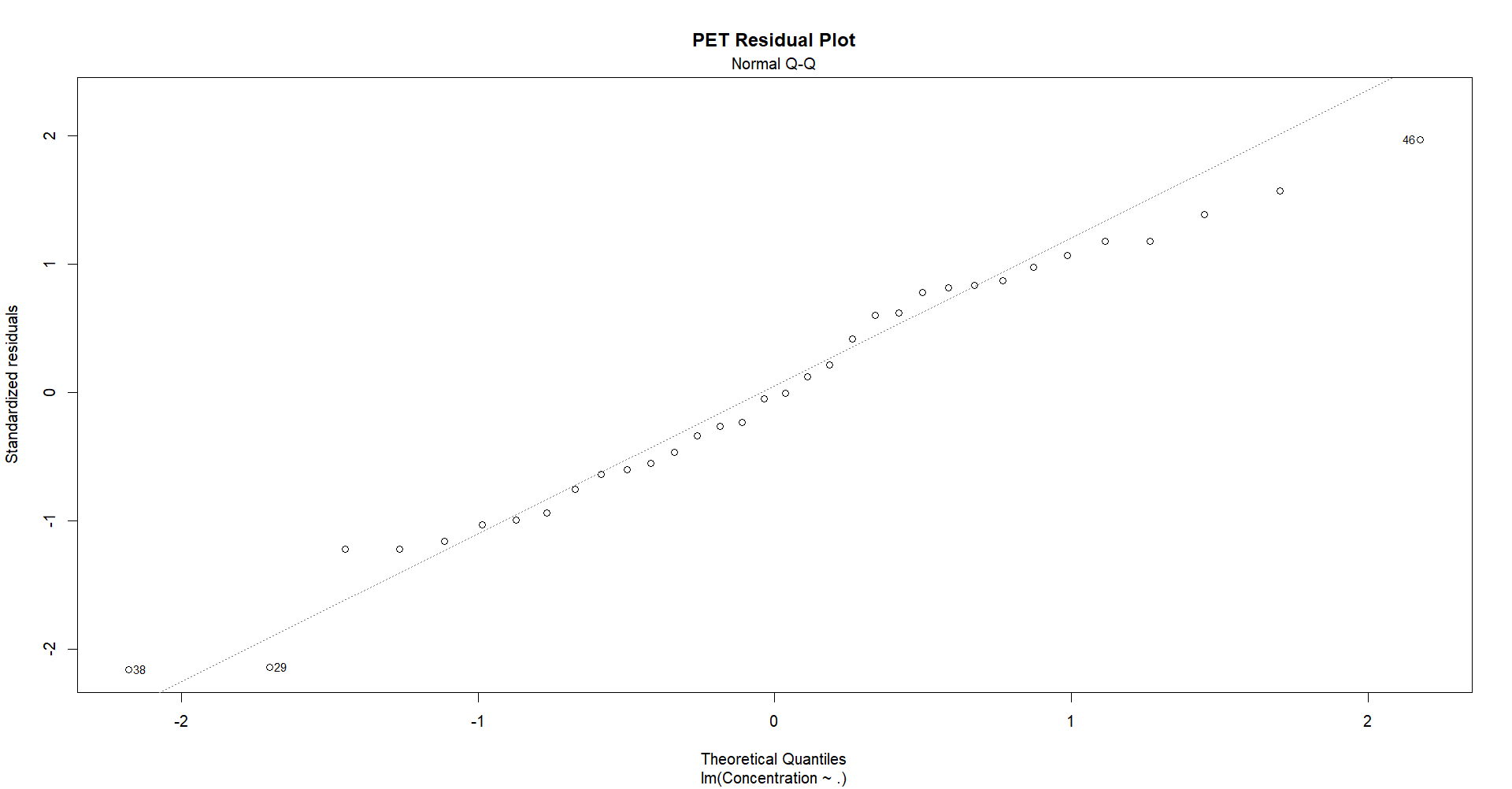


Fig. S5 (a): Normal Q-Q plot of PET


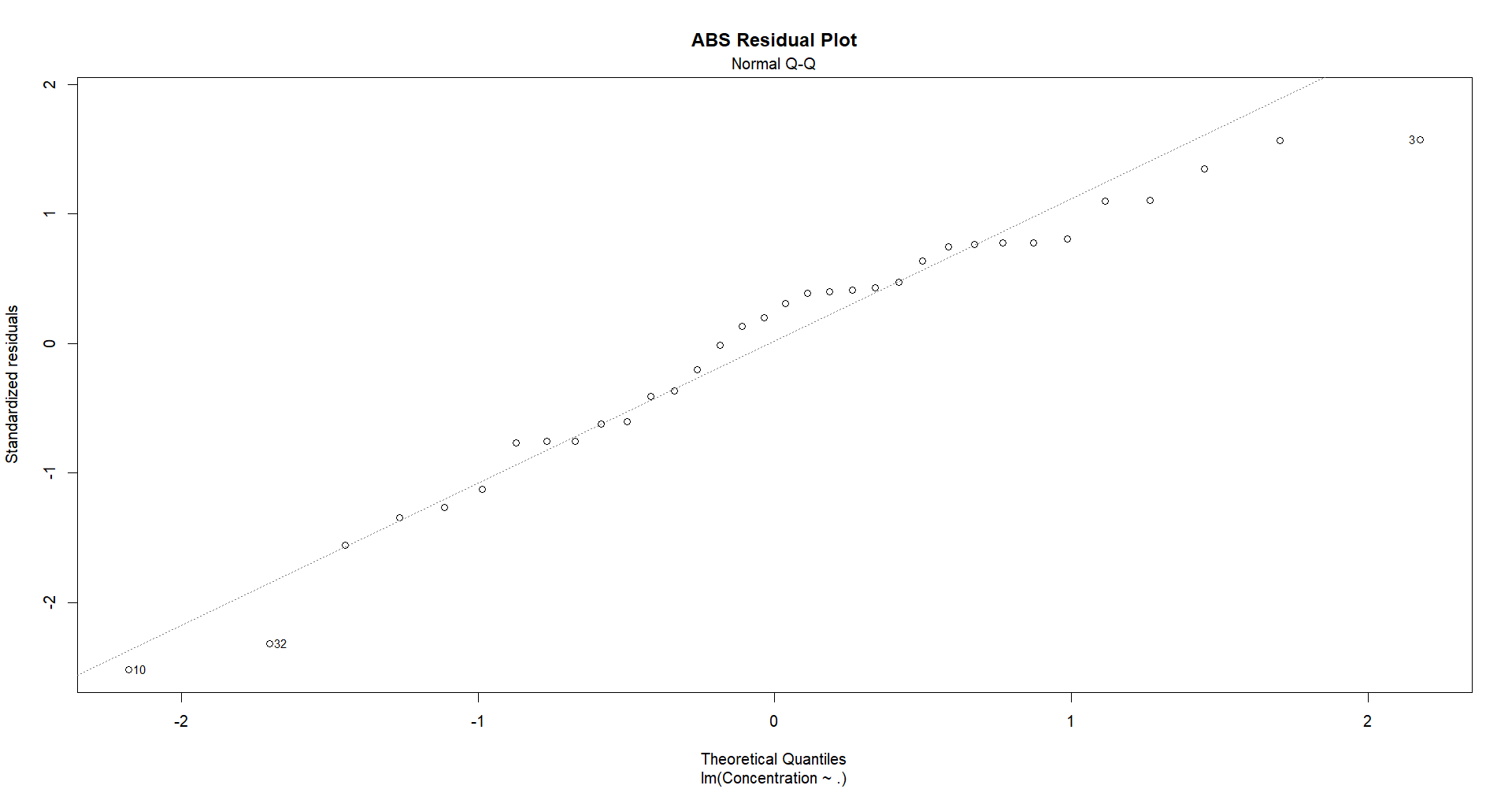


Fig. S5 (c): Normal Q-Q plot of ABS

**Fig S5 (a), (b) and (c):** **The Normal Q-Q plots of the trained models using the significant wavelengths of each microplastic dataset.** Normal Q-Q plots help determining if the residuals are normally distributed. If normally distributed, the point should roughly fall on the reference line.
